# Factors affecting the incidence of postoperative periprosthetic fractures following primary and revision hip arthroplasty: a systematic review and meta-analysis

**DOI:** 10.1186/s13018-020-02152-0

**Published:** 2021-01-06

**Authors:** Christos Bissias, Angelos Kaspiris, Athanasios Kalogeropoulos, Konstantinos Papoutsis, Nikolaos Natsioulas, Konstantinos Barbagiannis, Panayiotis J. Papagelopoulos, Olga D. Savvidou

**Affiliations:** 1 Department of Orthopaedic Surgery, Naval Hospital of Athens, Deinokratous 70, 115 21 Athens, Greece; 2 Laboratory of Molecular Pharmacology/Division for Orthopaedic Research, School of Health Sciences, University of Patras, 26504 Patras, Greece; 3 Sonnenhof Spital, Bern, Switzerland; 4 1st Department of Orthopaedic Surgery, School of Medicine, National and Kapodistrian University of Athens, “ATTIKON” University General Hospital, 124 62 Athens, Greece

**Keywords:** Risk factors, Periprosthetic fractures, Hip arthroplasty

## Abstract

**Objectives:**

The increasing number of hip arthroplasties (HA), due to the growing elderly population, is associated with the risk of femoral periprosthetic fractures (FPFs). The purpose of this study was to identify potential risk factors for the development of FPFs after HA.

**Methods:**

A systematic review was conducted in five data bases (Medline, Embase, Cochrane, Cinahl, ICTRP) according to the Preferred Reporting Items for Systematic reviews and Meta-analysis (PRISMA) guidelines up to May 2019, using the key words “risk factor,” “periprosthetic fracture,” and “hip replacement or arthroplasty.” Meta-analysis of the clinical outcomes of HA and subgroup analysis based on the factors that were implicated in FPFs was performed.

**Results:**

Sixteen studies were included (sample size: 599,551 HA patients, 4253 FPFs, incidence 0.71%). Risk factors statistically associated with increased incidence of FPFs were female gender (+ 40%), previous revision arthroplasty surgery (× 3 times), and the presence of rheumatoid arthritis (× 2.1 times), while osteoarthritis (− 57%), cement application (− 59%), and insertion of Biomet (− 68%) or Thompson’s prosthesis (− 75%) were correlated with low prevalence of FPFs. Obesity, cardiac diseases, advanced age, bad general health (ASA grade ≥ 3), and use of Exeter or Lubinus prosthesis were not linked to the appearance of FPFs.

**Conclusion:**

This meta-analysis suggested that female gender, rheumatoid arthritis, and revision arthroplasty are major risk factors for the development of FPFs after a HA. In those patients, frequent follow-ups should be planned. Further prospective studies are necessary to clarify all the risk factors contributing to the appearance of FPFs after HA.

**Supplementary Information:**

The online version contains supplementary material available at 10.1186/s13018-020-02152-0.

## Introduction

Femoral periprosthetic fractures (FPFs) after total hip arthroplasty (THA) were first described by Horwitz and Lenobel in 1954 [1]. FPFs constitute a devastating complication that often results in poor clinical outcome. Diagnosis of FPFs is typically made by the combination of clinical appearance, history of injury, and conventional radiographic examination. The widely used Vancouver classification provides a reliable evaluation of FPFs based on the femoral anatomic location of the fracture and the presence of a well-fixed or loose component [1]. The incidence of FPFs after hip arthroplasties (HA) has been reported between 0.045 and 4.1% [2–4]. The increasing prevalence of FPFs is directly associated with the increasing frequency of primary or revision HAs [5].

Although FPFs were not correlated with a specific implantation procedure, it was reported that they occurred more frequently after the application of cementless HA [6]. Factors that also contributed to the development of FPFs were the (a) low preoperative quality of patients’ bone stock like osteolytic or osteoporotic defects, (b) accompanied reduced mechanical properties of the implant surrounding tissue, (c) absence of stability of the implanted prosthesis, and (d) presence of pericapsular pathological changes [5]. Furthermore, patients with increased age, poor American Society of Anaesthesiologists (ASA) score, dementia, limited mobilization, partial weight bearing, and substantive functional limitations during postoperative period appeared to have a much higher risk for FPFs, implant failure, and mortality [6]. FPFs after primary HA resulted following spontaneous or low-energy injury corresponding to 8% or 75%, respectively [7, 8]. Treatment and postoperative rehabilitation of these fractures are complicated and expensive and correlate to increased morbidity and mortality. Specifically, the mortality rate after FPFs was remarkably increased in patients who had undergone primary joint replacement corresponding to 11% during the first year post-operatively. We must highlight the fact that a delay greater than 2 days from admission to the time of surgery also increased the mortality rate at one year [9].

Therefore, it is crucial to determine the potential risk factors that are associated with FPFs after total HA and hemiarthroplasty. Identification of risk factors for FPFs not only bridges the gap between clinical and basic or translational science but also improves the surgical practice in operating room as surgeons may incorporate novel concepts in their surgical techniques [10, 11]. Furthermore, in complex surgical problems, like FPFs, it is deemed necessary to understand the disease process and to integrate new scientific findings [12]. However, literature regarding the qualitative analysis of the identification of such risk factors is limited and does not provide adequate evaluation of their impact on clinical practice. Moreover, the majority of the studies included qualitative synthesis of limited sample size and/or number of surveys [13, 14] and/or potential risk factors [15].

Despite the fact that multiple identified risk factors for FPF have been described in the international literature including older age, female sex, bone fragility disorders, and systematic diseases [5, 6], there is a lack of a comprehensive study with generalized statistics analyzing the association between FPF and risk factors in both primary and revision HA. The aim of our systematic review and meta-analysis was to provide an up-to-date summary of the incidence and odds ratio of FPFs after performing primary and revision HA and to establish the contribution of potential risk factors in the development of FPFs.

## Materials and methods

This study was performed according to the Preferred Reporting Items for Systematic Reviews and Meta-Analyses (PRISMA) guidelines [16]. PRISMA checklist was used for reporting of relevant items for this meta-analysis and was provided in the supplementary document (Supplementary Table 1) [16].

### Literature search

A systematic computer-based literature review search with predefined criteria was attained on 08 May 2019 in the following databases: PubMed (1947 to present), Cochrane Database of Systematic Reviews (1992 to present), Embase (1974 to present), Cinahl, and WHO International Clinical Trials Registry platform (ICTRP). Research methodology was based on the combination of the following terms: “factors [All Fields],” “risk [All Fields],” “periprosthetic fractures [All Fields],” and “hip replacement, hemiarthroplasty or arthroplasty [All Fields].” The entire electronic literature search was conducted independently by two authors (CB and ODS) and an experienced clinical librarian. Moreover, the two senior authors (CB and ODS) screened the titles and abstracts independently in order to identify studies examining the clinical outcomes after the application of HA. If there was a disagreement between them, the final decision was made by the senior author.

### Study eligibility

Studies that examined the risk factors, the outcome, and the incidence of post-operative FPFs after performing HA were identified. HA was defined as the replacement of all or part of the hip joint by a prosthetic implant [17]. FPFs were defined as the femoral fractures that took place above, below, or close to an implanted prosthesis stem [18]. Only full-text articles were eligible for inclusion. The inclusion criteria were (a) studies written in English language, (b) comparative studies assessing the application of primary and revision hip arthroplasties, (c) surveys concerning HA that were performed in human subjects, and (d) data on the risk factors for FPFs and the outcome should have been clearly given for each patient. No publication date limitations there were set.

Studies that examined primary or metastatic hip cancers treated with HA, or surveys without comparative results, or being written in a language other than English were excluded. Case reports, reviews, letters to the editor, expert opinions articles, studies concerning PFs of acetabulum, research with insufficient details about the type of intervention, the clinical outcome, and surveys without obtainable data, were excluded.

### Data extraction

All data of each study was assembled in a Microsoft Excel spreadsheet, classified per intervention and type of periprosthetic fracture. Characteristics extracted from clinical studies included authorship, publication year, study design (cohort or randomized control trial), single or multicenter status, enrolled sample number, population gender and age, and risk factors in both control and treatment groups, HA procedure, outcomes regarding the frequency of periprosthetic fracture development, and the type of HA. Data from each study are summarized in Table 1.

**Table 1:** Clinical characteristics of studies included in the meta-analysis

	Author, year	Country	Type of study	Number of patients	Number of periprosthetic fractures	Follow-up period (in months)	Risk factors
**1**	Sarvilinna et al. [19]	Finland	Prospective cohort study	31599	1555	144	Gender, prosthesis type, and age without significance as risk factors, Risk of PF was about the same in patients operated with or without the cemented prosthesis
**2**	Sarvilinna et al. [20]	Finland	Cases control study	31	31	N/A*	Fracture as the primary diagnosis, Protective factors: cemented prosthesis, Thompson prosthesis, and Biomet prosthesis, and they were associated with increased incidence of loosening of femoral component and reduced incidence of infection and dislocation
**3**	Sarvilinna et al. [21]	Finland	Cases control study	48	16	N/A*	Young age at the time of the hip fracture and polished wedge type of prosthesis, Protective factors: Thompson prosthesis and Biomet prosthesis
**4**	Berend et al. [22]	USA	Prospective cohort study	2551	59	81	Anterolateral approach, uncemented femoral fixation, and female sex, Protective factors: cemented prostheses but they were associated with reduced femoral component survivorship
**5**	Cook et al. [23]	U.K.	Case–control study	6334	124	204	Patients older than 70 years, cemented arthroplasties
**6**	Meek et al. [24]	U.K.	Prospective cohort study	51628	508	60	Female gender, age > 70 and revision arthroplasty
**7**	Zhang et al. [25]	China	Retrospective cohort study	424	26	N/A*	Cemented and revision arthroplasties, osteoporosis, and previous fracture
**8**	Savin et al. [26]	Romania	Retrospective cohort study	3574	47	N/A*	Cementless and revision arthroplasties, Protective factors: cemented prosthesis
**9**	Singh et al. [27]	USA	Prospective cohort study	5951	330	67	Female gender, high Deyo-Charlson comorbidity index, and revision arthroplasties
**10**	Singh et al. [28]	USA	Prospective cohort study	13760	305	75	Female gender, high Deyo-Charlson comorbidity index, ASA score ≥ 2, and cemented arthroplasties
**11**	Katz et al. [29]	USA	Prospective cohort study	31443	215	156	Older age and female gender
**12**	Thien et al. [30]	Sweden	Prospective cohort study	436861	768	24	Shape and surface finish of the femoral stem and cemented arthroplasties, Protective factors: cemented prostheses but they were associated with higher risk of FPFs in male compared with female patients
**13**	Ricioli Jr et al. [31]	Brazil	Retrospective cohort study	1771	101	180	Female gender aged ≥ 65 years, presence of a previous hip surgery, and revision arthroplasties
**14**	Gromov et al. [32]	Denmark	Retrospective cohort study	1550	48	24	Bone morphology (femoral Dorr type C), female gender, and cementless prosthesis
**15**	Lindberg-Larsen et al. [33]	Denmark	Prospective cohort study	7019	150	03	Uncemented femoral stem, medically treated osteoporosis, female sex, and older age, Protective factors: cemented prosthesis
**16**	Tamaki et al. [34]	Japan	Retrospective cohort study	833	17	03	Short stem length and cementless prosthesis

*Not applicable

*FPFs* femoral periprosthetic fractures

Two reviewers (CB and ODS) examined all the identified surveys and extracting data by using a predetermined form. The presence of duplicate studies was examined using Endnote software (Clarivate Analytics, Philadelphia, Pennsylvania, USA).

### Study selection and quality assessment

The methodology of each study was assessed independently by the two senior authors (CB and ODS) using the Newcastle–Ottawa quality assessment scale [35]. Included studies were graded according to a three-category scale. Surveys that appeared a total score of 0–3, 4–6, and 7–9 were classified to be of a poor, fair, or good quality, respectively (Table 2a). Modified Jadad scale for clinical trials was also used to evaluate the quality of included trials [36]. Jadad score greater than 4 was considered to be of high quality (Table 3). There were not exclusion criteria for age, population, diagnosis, or quality of the studies. Funnel plots were built in order to determine the aspect of publication bias that may affect the conclusions of our analysis.

**Table 2:** Study quality of the included studies based on the Newcastle–Ottawa scale

	Author, year	Representativeness of the exposed cohort	Selection of the nonexposed cohort	Ascertainment of exposure	Demonstration that outcome of interest was not present at start of the study	Comparability of cohorts on the basis of the design or analysis	Assessment of the outcome	Follow-up long enough for outcomes*	Adequacy of follow-up of cohort**	Total	Quality
**1**	Sarvilinna et al. [19]	1	1	1	1	2	1	1	1	09	Good
**2**	Sarvilinna et al. [20]	1	1	1	1	2	1	0	0	08	Good
**3**	Sarvilinna et al. [21]	1	1	1	1	2	1	0	0	08	Good
**4**	Berend et al. [22]	1	1	1	1	2	1	1	1	09	Good
**5**	Cook et al. [23]	1	1	1	1	2	1	1	1	09	Good
**6**	Meek et al. [24]	1	1	1	1	2	1	0	1	08	Good
**7**	Zhang et al. [25]	1	1	1	1	2	1	0	0	07	Good
**8**	Savin et al. [26]	1	1	1	1	2	1	0	0	07	Good
**9**	Singh et al. [27]	1	1	1	1	2	1	1	1	09	Good
**10**	Singh et al. [28]	1	1	1	1	2	1	1	1	09	Good
**11**	Katz et al. [29]	1	1	1	1	2	1	1	1	09	Good
**12**	Thien et al. [30]	1	1	1	1	2	1	0	1	08	Good
**13**	Ricioli Jr et al. [31]	1	1	1	1	2	1	1	1	09	Good
**14**	Gromov et al. [32]	1	1	1	1	2	1	0	1	08	Good
**15**	Lindberg-Larsen et al. [33]	1	1	1	1	2	1	0	0	07	Good
**16**	Tamaki et al. [34]	1	1	1	1	2	1	0	0	07	Good

^*^Follow-up more than 24 months

^**^Lost to follow-up rate more than 10% is considered inadequate

**Table 3:** Study quality of the included studies based on the modified Jadad scale

	Author, year	Randomization	Concealment of allocation	Double blinding	Total withdrawals and dropouts	Total	Quality
**1**	Sarvilinna et al. [19]	**	*	*	**	06	Good
**2**	Sarvilinna et al. [20]	*	*	*	*	04	Good
**3**	Sarvilinna et al. [21]	*	*	*	*	04	Good
**4**	Berend et al. [22]	**	*	*	**	06	Good
**5**	Cook et al. [23]	**	*	*	*	05	Good
**6**	Meek et al. [24]	**	*	*	**	06	Good
**7**	Zhang et al. [25]	*	*	*	*	04	Good
**8**	Savin et al. [26]	*	*	*	*	04	Good
**9**	Singh et al. [27]	**	*	*	**	06	Good
**10**	Singh et al. [28]	**	*	*	**	06	Good
**11**	Katz et al. [29]	**	*	*	**	06	Good
**12**	Thien et al. [30]	**	*	*	**	06	Good
**13**	Ricioli Jr et al. [31]	*	*	*	*	04	Good
**14**	Gromov et al. [32]	*	*	*	*	04	Good
**15**	Lindberg-Larsen et al. [33]	**	*	*	**	06	Good
**16**	Tamaki et al. [34]	*	*	*	*	04	Good

*Indicates one point

**Indicated two points

### Statistical analysis

This meta-analysis was conducted in line with the recommendations from Cochrane and PRISMA statement [16]. Statistical analysis was performed with the STATA Statistical software, version 11.0 (Stata Corp LLC, College Station, TX, USA). The incidence of FPFs after the application of HAs, the correlated risk factors and the odds ratios (ORs), and the associated 95% confidence intervals (95% CI) were calculated. Heterogeneity between the trials was calculated by using Cochrane *Q* and the inconsistency (*I*^*2*^) test. The degree of heterogeneity was graded as low (*I*^2^ < 25%), moderate (*I*^2^ from 25 to 75%), and high (*I*^2^ > 75%). A random effect model was used to calculate pooled ORs in the case of significant heterogeneity, while the fixed effect model was used in the studies with low heterogeneity. This was undertaken because in sensitivity analysis the presentation of both models provides comprehensive evaluation of how differences in datasets affected the observed outcomes [37]. Egger’s test and graphical exploration with funnel plots were used to evaluate the risk of publication bias. The level of statistical significance was defined as *p* < 0.05.

## Results

### Study characteristics

**Fig. 1:**
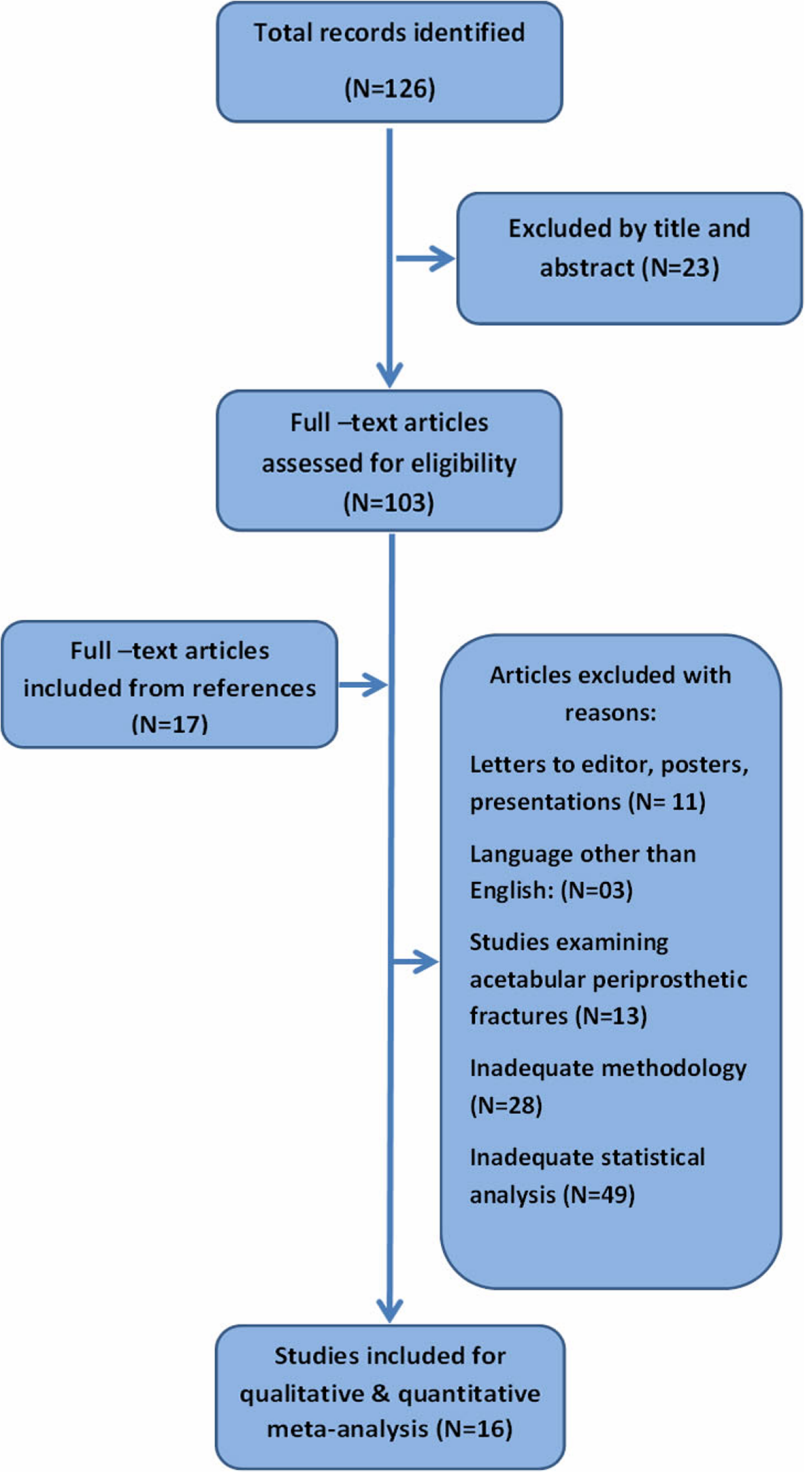
Preferred Reporting Items for Systematic Reviews and Meta-Analysis (PRISMA) flowchart for the screening and identification of included studies

In the initial search, a total of 126 relevant trials were detected. After the initial evaluation of the studies based on the abstract and title, 103 publications were included. The further analysis of the remaining papers resulted in the exclusion of 104 surveys. Twenty-eight studies were excluded due to inadequate methodology, while 49 studies were declined being statistically unsatisfactory. Moreover, 13 studies examined acetabular PFs, 3 were written in other language than English and 11 surveys were not original studies and were analyzed in the flowchart of Fig. 1.

Finally, sixteen studies published between 2003 and 2018 met our inclusion criteria for the analysis of potential risk factors for the development of FPFs after THA [19–34]. The grade of the agreement among the reviewers that evaluated the scientific quality of the included studies was strong. The main characteristics of the included participants are displayed in the Table 1.

### Quality assessment

In Tables 2 and 3, the methodological quality of the enrolled studies is summarized. According to the Newcastle–Ottawa scale and the modified Jadad score, all the enrolled trials were considered being of good and high quality and, therefore, were judged to be at a low risk of bias.

Furthermore, funnel plots were created to evaluate publication bias. After this evaluation, all studies were found to lie within a 95% CI as represented by the inverted funnel, suggesting absence of publication bias.

### Outcomes

**Fig. 2:**
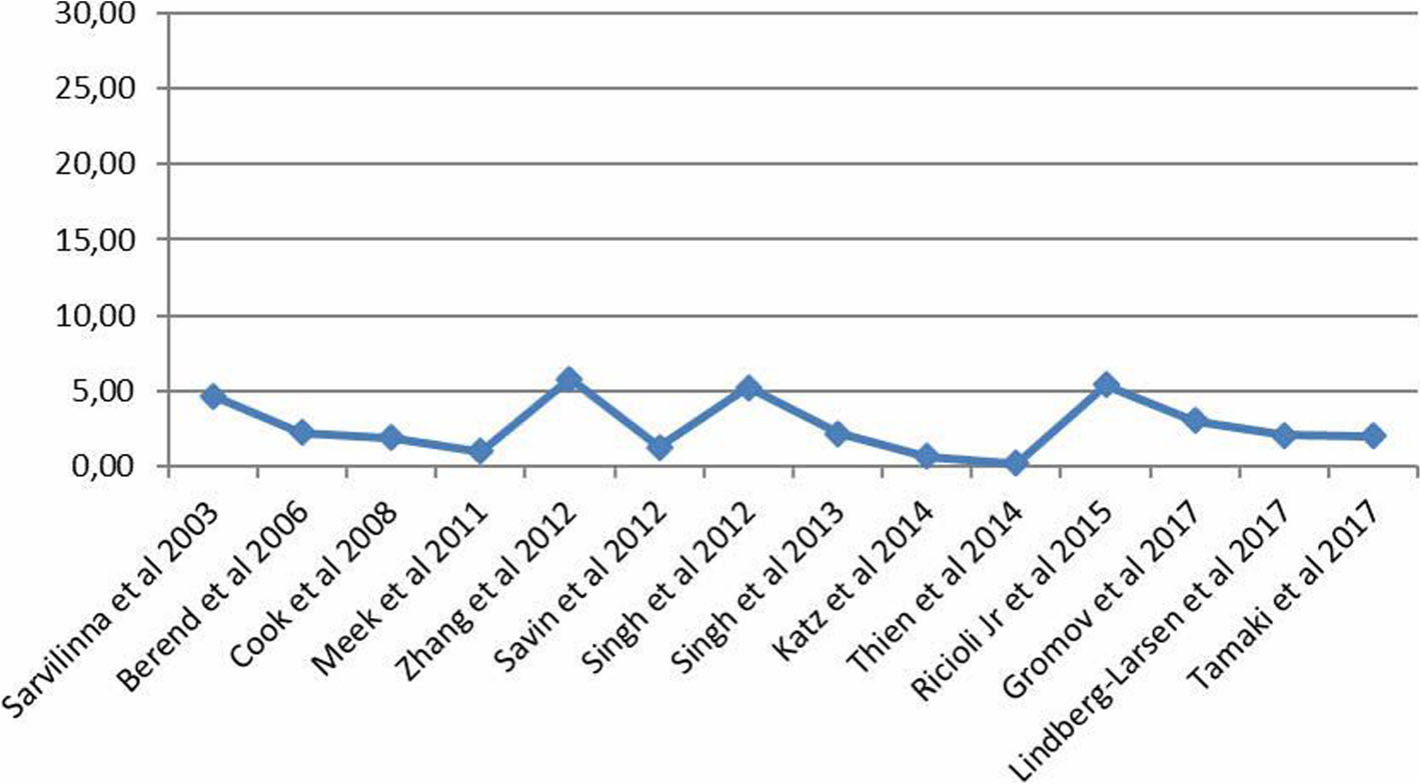
Diagram demonstrating the incidence of the collected reported femoral periprosthetic fractures after hip arthroplasty between 2003 and 2017

In total, 599.677 HA were included in the meta-analysis. Fourteen studies were used to reveal the prevalence of FPFs [20, 22–34], as two of the studies were characterized as case–control surveys [19, 21] and were excluded of prevalence calculation. Finally, 599.551 HA and 4253 FPFs were reported, demonstrating prevalence of 0.71%. In specific, the reported cases between 2003 and 2013 corresponded to incidence of 2.4%, whereas the prevalence of FPFs between 2014 and 2018 was reduced to 0.27% (Fig. 2).

### Epidemiological risk factors

**Fig. 3:**
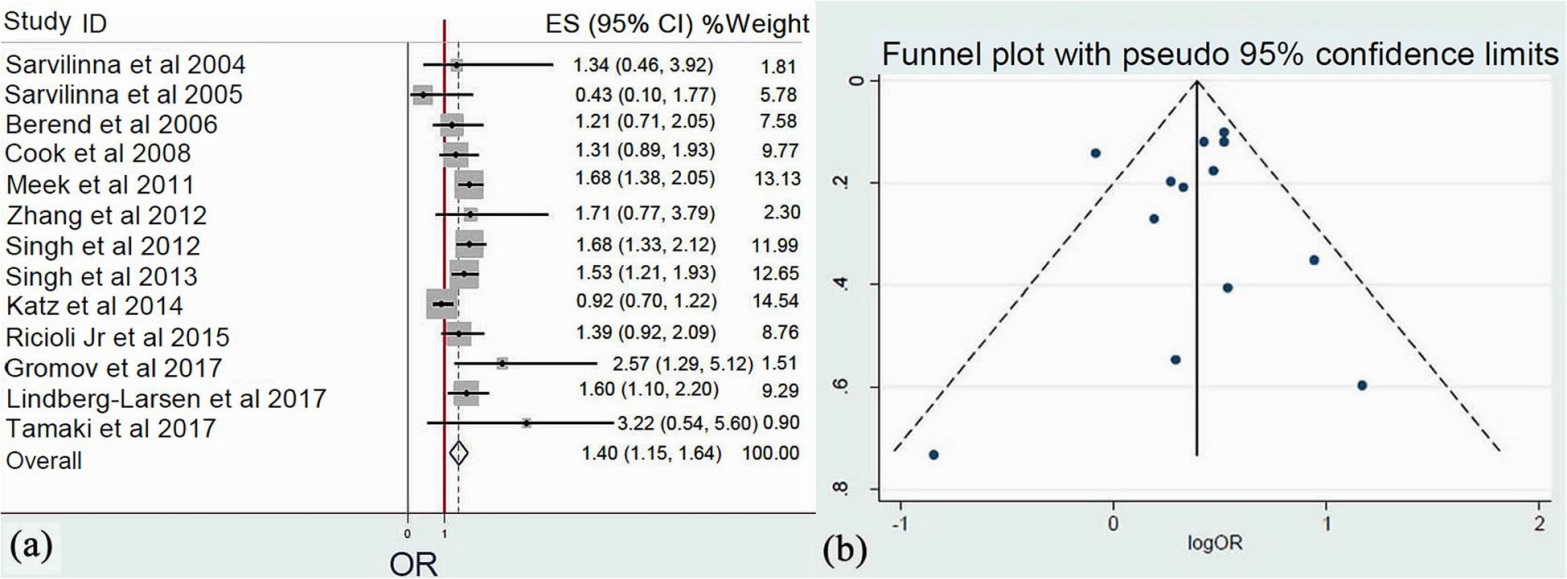
**a** Forest plot demonstrating the female gender as a significant risk factor for femoral periprosthetic fractures after hip arthroplasty. **b** Funnel plot demonstrating the low risk of publication bias of the included studies

Thirteen studies [20–25, 27–29, 31–34] analyzed gender as a risk factor for the development of femoral PFs after HA. Meta-analysis of these studies (*Q* = 27.4, *I*^2^ = 56.2%, *P* = 0.007) revealed that female gender was as much as 40% more likely to sustain FPFs (OR: 1.40, 95% CI: 1.15–1.64, *p* < 0.001) (Fig. 3).

Five studies [20, 23, 27–29] and six studies [20, 23, 24, 27, 28, 32] reported age greater than 80 years and 70 years as a risk factor, respectively. Meta-analysis of these studies (*Q* = 18.4, *I*^2^ = 78.2%, *P* = 0.765, and *Q* = 59.1, *I*^2^ = 91.5%, *P* = 0.001, respectively) revealed that age (older than 80 and 70 years) was not a significant risk factor for the development of FPFs (OR: 1.36, 95% CI: 0.79–1.94, *p* = 0.249, and OR: 1.31, 95% CI: 0.82–1.81, *p* = 0.351, respectively).

Obesity was examined as a risk factor for FPF appearance in four surveys [27, 28, 32, 33]. Meta-analysis of these trials (*Q* = 0.17, *I*^2^ = 0.0%, *p* = 0.982) demonstrated that obesity was not an important risk factor associated with increased frequency of FPFs (OR: 0.90, 95% CI: 0.76–1.03, *p* = 0.164).

### General medical condition risk factors

In three studies [21, 27, 28], general health status of the patients who underwent HA was evaluated with the ASA Physical Status Classification System. However, meta-analysis of these studies (*Q* = 11.3, *I*^2^ = 81.9%, *p* = 0.001) did not identify the ASA score ≥ 3 as statistically significant risk factor for FPF appearance (OR: 0.47, 95% CI: 0.43–1.31, *p* = 0.731).

Similarly, meta-analysis (*Q* = 4.56, *I*^2^ = 56.1%, *p* = 0.102) of three studies [21, 28, 29], which explored pre-existing cardiac disease as a risk factor for FPF development, showed that heart pathology was not associated with increased incidence of FPFs (OR: 1.00, 95% CI: 0.65–1.36, *p* = 0.289).

### Joint diseases risk factors

**Fig. 4:**
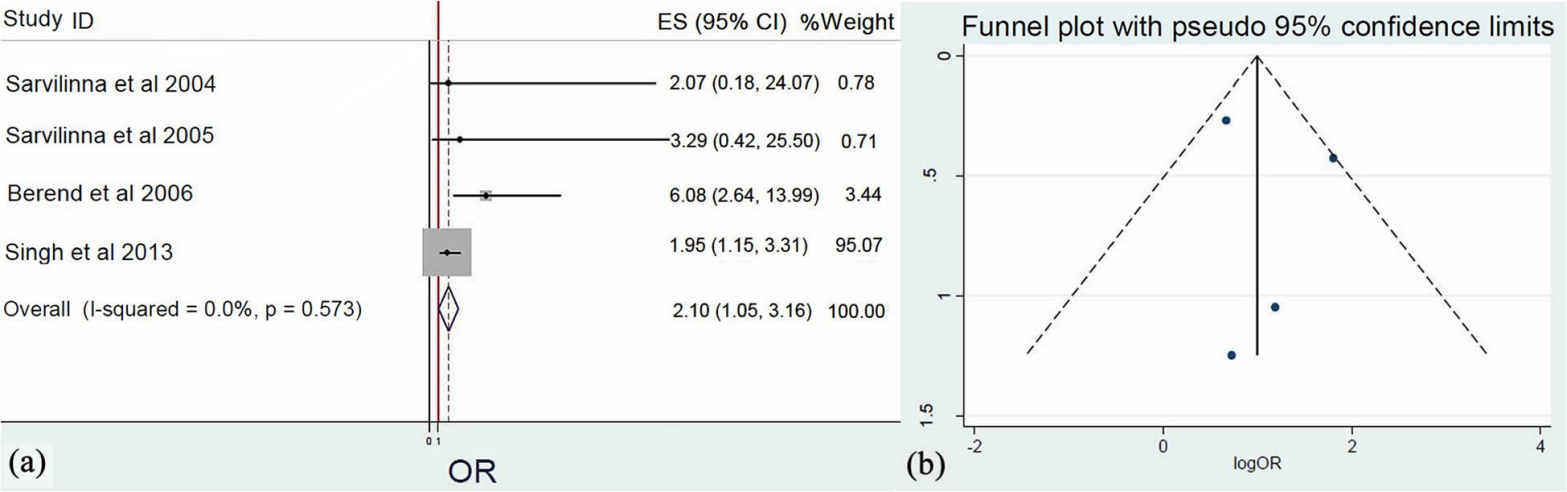
**a** Forest plot demonstrating rheumatoid arthritis as a significant risk factor for femoral periprosthetic fractures after hip replacement. **b** Funnel plot demonstrating the low risk of publication bias of the included studies

Meta-analysis (*Q* = 2.0, *I*^2^ = 0.0%, *p* = 0.573) of four surveys [20–22, 28] that included the examination of rheumatoid arthritis (RA) as a risk factor for PFs demonstrated that RA is a remarkable risk factor contributing in FPF development. Specifically, patients with RA who underwent HA had 2.1 times greater risk to experience FPFs (OR: 2.1, 95% CI: 1.05–3.15, *p* = 0.009) (Fig. 4).

**Fig. 5:**
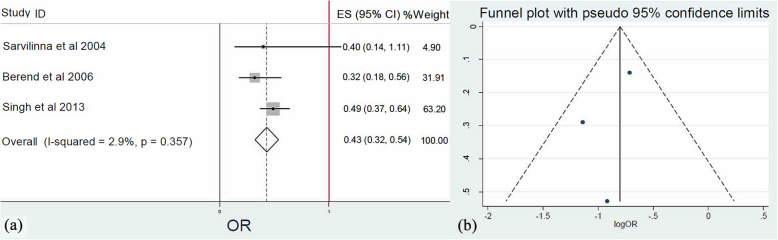
**a** Forest plot demonstrating osteoarthritis as a significant protective factor for femoral periprosthetic fractures after hip arthroplasty. **b** Funnel plot demonstrating the low risk of publication bias of the included studies

Meta-analysis (*Q* = 2.06, *I*^2^ = 2.09%, *p* = 0.357) of three studies [20, 22, 28] that reviewed osteoarthritis (OA) as potential factor implicated in FPF evolvement revealed that OA was negatively associated with the appearance of FPFs. Indeed, patients with OA had 57% reduced risk to experience FPFs (OR: 0.43, 95% CI: 0.32–0.54, *p* = 0.010) (Fig. 5).

### Fixation and implant type risk factors

**Fig. 6:**
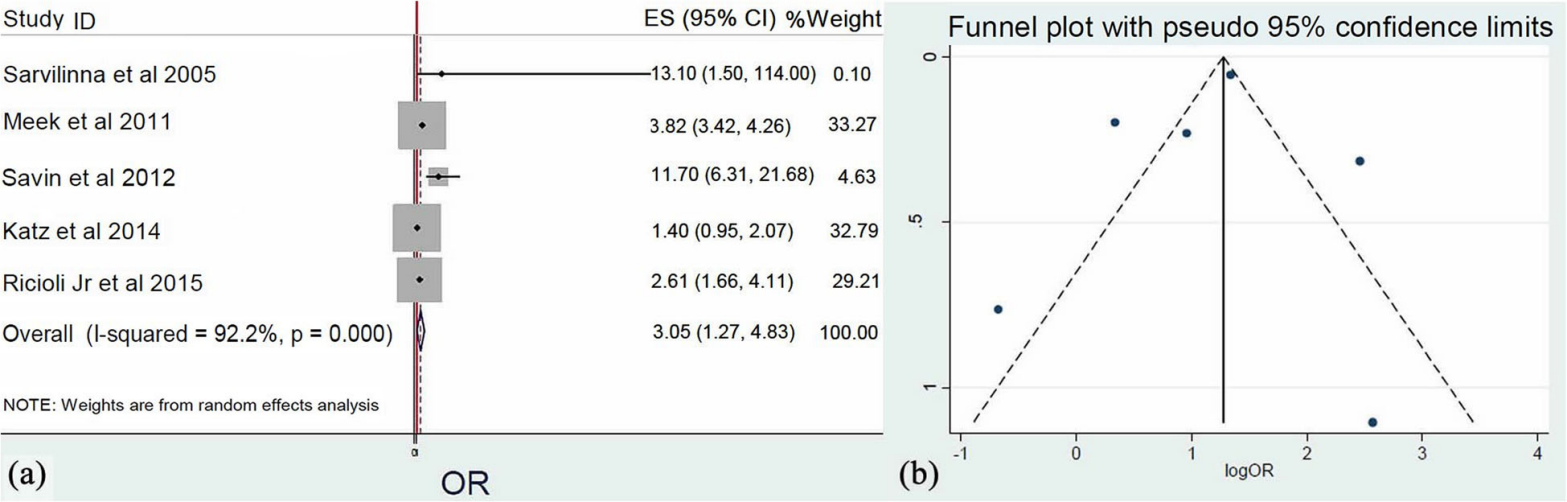
**a** Forest plot demonstrating revision hip arthroplasty as a significant risk factor for femoral periprosthetic fractures. **b** Funnel plot demonstrating the low risk of publication bias of the included studies

In five studies [21, 24, 26, 29, 31], the presence of a revision HA was reported. Meta-analysis (*Q* = 51.3, *I*^2^ = 91.2%, *p* < 0.001) of these studies identified that the risk of FPFs after revision HA is 3 times higher than primary HA (OR: 3.05, 95% CI: 1.27–4.82, *p* = 0.005) (Fig. 6).

**Fig. 7:**
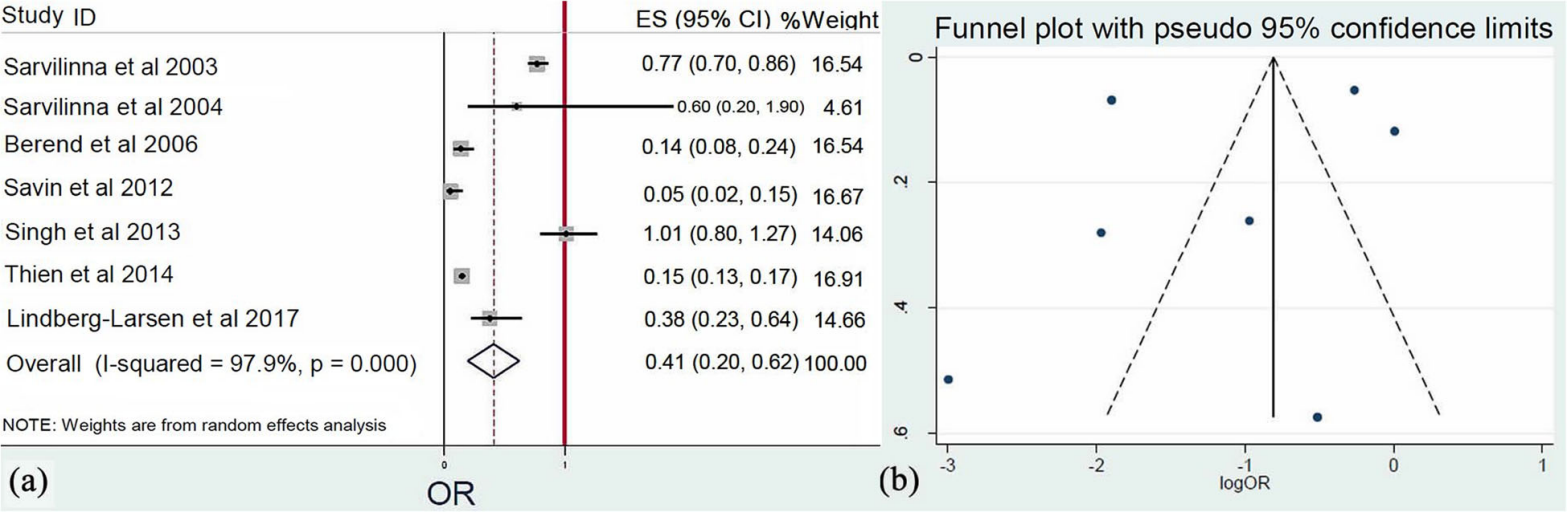
**a** Forest plot demonstrating the insertion of cemented prosthesis as a significant protective factor for femoral periprosthetic fractures. **b** Funnel plot demonstrating the low risk of publication bias of the included studies

In seven studies [19, 20, 22, 26, 28, 30, 33], the application of cemented prosthesis was presented. Meta-analysis of these surveys (*Q* = 286.4, *I*^2^ = 97.9%, *p* = 0.001) confirmed that the use of cemented femoral prosthesis was a significant protective factor, decreasing the possibility of FPFs (0.41, 95% CI: 0.19–0.62, *p* < 0.001) (Fig. 7).

**Fig. 8:**
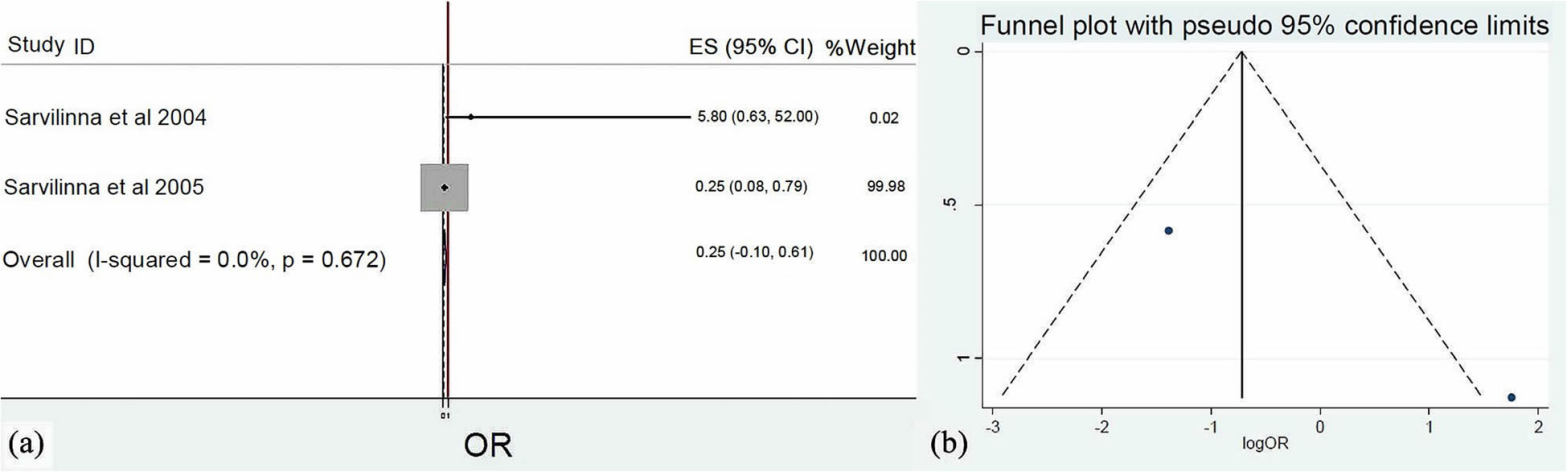
**a** Forest plot demonstrating the application of Thompson prosthesis as a significant protective factor for femoral periprosthetic fractures after hip arthroplasty. **b** Funnel plot demonstrating the low risk of publication bias of the included studies

**Fig. 9:**
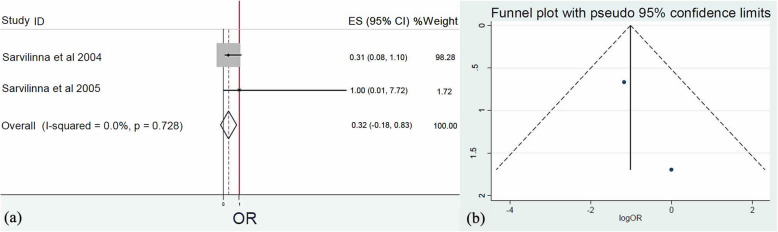
**a** Forest plot demonstrating the application of Biomet prosthesis as a significant protective factor for femoral periprosthetic fractures after hip arthroplasty. **b** Funnel plot demonstrating the low risk of publication bias of the included studies

The impact of the implant type that was inserted during THA was also evaluated. Meta-analysis of four [19–21, 23], two [20, 21], three [19–21], and two [20, 21] studies regarding Exeter (Stryker, Kalamazoo, USA) (*Q* = 17.1, *I*^2^ = 82.5 %, *p* = 0.001), Thompson (Stryker UK Ltd., Newbury, UK) (*Q* = 0.18, *I*^2^ = 0.0%, *p* = 0.672), Lubinus (Waldemar Link GmbH & Co, Hamburg, Germany) (*Q* = 0.0, *I*^2^ = 0.0%, *p* > 0.999), and Biomet (Zimmer Biomet Holdings, Warsaw, USA) (*Q* = 0.0, *I*^2^ = 0.0%, *p* > 0.999) prosthesis, respectively, indicated that Thompson (OR: 0.25, 95% CI: 0.00–0.61, *p* = 0.010) (Fig. 8) and Biomet (OR: 0.32, 95% CI: 0.00–0.83, *p* = 0.021) (Fig. 9) prostheses were associated with reduced risk of FPFs. Conversely, Exeter (OR: 0.97, 95% CI: 0.03–1.91, *p* = 0.759) and Lubinus (OR: 1.02, 95% CI: 0.90–1.14, *p* = 0.869) implants did not favor the development of FPFs.

## Discussion

Femoral periprosthetic fractures after HA constitute a major complication and are usually associated with increased mortality rate and inadequate functional recovery [38]. However, the general and local risk factors that contribute in the development of FPFs remain relatively unclear. This meta-analysis focused on the examination of the incidence and the detection of possible predisposing factors leading to FPFs.

Our analysis indicated that the prevalence of FPFs after HA was 0.71%. Interestingly, during the last 4 years the frequency of FPFs was limited to 0.27%. The reported incidences range from 0.3 to 27.8% after primary HA and from 0.3 to 17.6% after revision HAs [3, 39, 40]. Despite the fact that a growing rate of HA was noted [39], the incidence of FPFs was reduced. The progressive identification of risk factors, the advancing orthopedic education in hip surgical techniques, and the ongoing surgical experience may explain the above finding.

The frequency of FPFs was higher after the insertion of uncemented HA ranging from 3 to 18% [14, 40, 41]. This is in line with our findings, where cemented prosthesis was a protective factor for FPFs. Our observations confirmed Berry’s report who observed an increased frequency of 20.9% for intra-operative fractures when using of uncemented fixation, compared with 3.6% for cemented femoral revisions [3]. Similarly, only a 3% of intra-operative FPFs were noted after the insertion of cemented implants [42], being three times less common compared with uncemented stems [43]. The above result may be explained by the fact that the insertion of cement into a weak osteoporotic femur stabilizes the bony structure and enhances the bone biomechanical properties [40]. Additionally, failure of the bone biological ingrowth or ongrowth process or occurrence of a femoral crack and microfracture during the insertion of a press-fit cementless prosthesis may lead to increased rate of FPFs even after low-energy trauma. Although the impact of implant type has been examined in many surveys, the results vary from study to study [22, 23]. Inadequate surgical familiarity with the uncemented technique and different methodological protocols (e.g., population samples or follow-up duration) could be correlated with these discrepancies.

Based on our results, revision HA was strongly associated with FPFs, a consistent result in many surveys [3, 14, 44, 45]. The insertion of a new femoral stem in a weak proximal femur due to excessive osteopenia or osteoporosis, accompanied by concurrent development of intra-operative stress shielding or cortical injuries during the removal process of the previous stem or cement and the application of longer or larger-diameter stems, especially using reaming procedure, may provide an explanation for the above findings [44, 45].

Little is known about the association between femoral implant design characteristics and the frequency of FPFs. Our results did not confirm the previously reported correlation of Exeter prosthesis to increased rate of FPFs [46]. Conversely, insertion of Exeter and Lubinus prostheses did not increase the prevalence of FPFs, whereas Thompson and Biomet implants significantly decreased the incidence of FPFs (by 75% and 68%, respectively). Sarvilinna et al. reported that in patients with hip fractures treated with HA, the polished wedge type of prosthesis was linked to an increased risk of FPFs [21]. Results from a large Norwegian Hip Fracture Register, which were undertaken in patients with a femoral neck fracture, demonstrated high re-operation ratio due to FPFs after the application of polished stems compared to anatomical and straight stems [47]. This is, also, consistent with a UK National Joint Registry study which investigated revision interventions for FPFs after THAs and found lower incidence of FPFs with a Charnley stem [48]. Finally, a large retrospective cohort study conducted by the UK National Joint registry after the analysis of 299,019 primary THAs reported that the high rate of FPFs after the insertion of polished hip cemented stems was, also, associated with cobalt-chromium stem material, the increased stem offset, the ovaloid and round diaphyseal cross-sectional stem shape, and the increased head size [49].

Our results showed that OA was a protective factor in FPF appearance, while RA was a significant risk factor being in line with previous studies [4, 14, 40, 44, 45]. Poor bone quality, multiple joint involvement and considerable comorbidity, may explain why the presence of RA was associated with a high risk of FPFs. Furthermore, the significant bone erosion, osteolytic defects, and the simultaneous induced expression of osteoclasts and inflammatory cytokines may result in the generation of FPFs [50]. Clinical studies also confirmed the close association between low Bone Mineral Density (BMD) and RA leading to increased bone loss and femoral frailty [51, 52]. It was suggested that prevention of late FPFs could be accomplished by the intra-operative recognition of locations of cortical defects and osteolytic lesions and the prophylactically application of cortical grafts to reinforce cortical weakness and other stress risers [15]. Contrariwise, the exact mechanism of OA-protective effects in the appearance of FPFs is largely unknown. Patients with OA are characterized by reduced level of activity due to localized arthralgia and limitation of joint movements, especially to those who were overweight. Furthermore, altered embiomechanical bony structure due to subchondral sclerosis and absence osteoporotic defects may provide an extra explanation of this finding in OA [53].

Based on our findings, female gender was an important epidemiological factor that increased the risk of FPFs by 40%. Although female gender has been suggested to be an independent risk factor, it is obviously confounded by osteoporosis [45]. Contrariwise, age older than 70 or 80 years, obesity, medical comorbidities such as cardiac disease, or physical condition with ASA score ≥ 3 were not related with high rate of FPFs, confirming the results of previous studies [14, 40].

## Strengths and limitations

Strength of our analysis was that it included all the current international literature comprising a large number of prospective studies and a large population sample (8 times larger than in previous analyses) [14, 15]. However, the selected studies had the following limitations: First, the prevalence of FPFs was calculated by a pool that included both hemiarthroplasties and THAs patients. However, in the international literature, a large number of studies that examined the risk factors, the outcomes, and the frequency of FPFs have enrolled patients of both interventions [54, 55]. Moreover, the fact that the mean incident of complications, including FPFs, did not differ significantly between patients treated with hemiarthroplasty or THAs [4, 56] does not alter the credibility of our results. Another limitation may was the fact that only studies written in English were reviewed and thus some studies may be missing in the analysis. Nevertheless, the vast majority of critical reviews and meta-analyses on the international literature follow the same methodology. Additional drawbacks could be the heterogeneity of data population, the variability of diagnostic and treatment protocols, the different selection criteria and follow-up periods, and the absence of diseases severity classification (e.g., in OA). Finally, other limitation factors were the differences in methodological approaches and the conditions under which the studies were conducted or other confounding factors that were not taken into consideration.

## Conclusions

This meta-analysis suggested that female gender, RA, and revision arthroplasty are major risk factors for the development of FPFs whereas OA, cement application, and insertion of Biomet or Thompson’s prosthesis were correlated with low prevalence of FPFs. Obesity, cardiac diseases, advanced age, poor general health (ASA grade ≥ 3), and use of Exeter or Lubinus prosthesis did not conduce to the appearance of FPFs. Based on the meta-analysis data, it could be recommended that (a) insertion of a femoral implant designed with anatomic characteristics can reduce the risk of re-operation in patients of similar age, sex, and bone quality; (b) intra-operative application of cortical grafts may prevent possible bone defects or stress risers in patients with known risk factors like RA or revision surgery [15]; (c) cement insertion for the fixation of the femoral implant is suggested to reduce the risk of FPFs; (d) systematic clinical and radiographic postoperative follow-ups are necessary to examine the stem stability and bone quality; and (e) the pre- and postoperative nutritional status and BMD level must be assessed and corrected, especially in patients suffering of RA. However, the risk of atypical femoral neck fractures after prolonged bisphosphonate therapy should be considered [57].

Future basic science and clinical prospective studies are warranted to establish stronger evidence regarding the mechanisms that alter bone strength and quality in female patients and in those suffering of rheumatic diseases resulting in FPFs, to strengthen the efficacy of insertion of Biomet or Thompson’s prostheses though cemented procedure in the prevention of FPFs and to produce more robust results for the clarification of the potential risk factors contributing to the development of FPFs after HAs.

## Supplementary Information

**Additional file 1: Supplemental Table 1**. PRISMA checklist

## Data Availability

Datasets are available through the corresponding author upon reasonable request

## Abbreviations

ASA: American Society of Anaesthesiologists
95% CI: 95% confidence intervals
FPFs: Femoral periprosthetic fractures
HA: Hip arthroplasties
ICTRP: International Clinical Trials Registry platform
OA: Osteoarthritis
ORs: Odds ratios
PRISMA: Preferred Reporting Items for Systematic Reviews and Meta-Analysis
RA: Rheumatoid arthritis
THA: Total hip arthroplasty
WHO: World Health Organization
